# Supplementary figures and images for: Neural activity during a simple reaching task in macaques is counter to gating and rebound in basal ganglia–thalamic communication
Source: PLoS Biol. 2020 Oct 13;18(10):e3000829. doi: 10.1371/journal.pbio.3000829 (PMC7584254; doi:10.1371/journal.pbio.3000829)

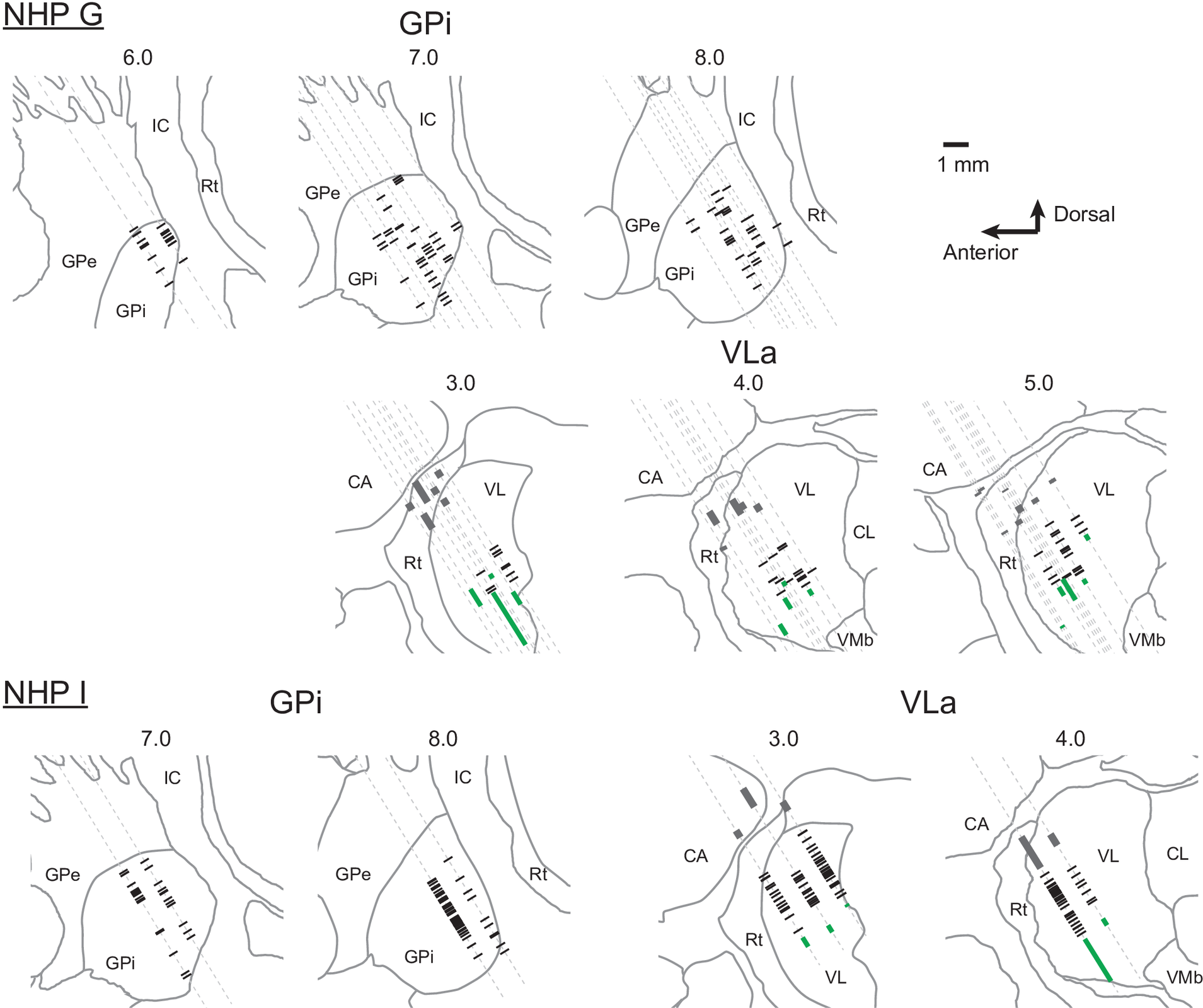

Supplement: S1 Fig — Green bars indicate the locations of SCP-responsive VLp neurons. Gray bars indicate the locations of activity characteristic of the reticular nucleus of the thalamus. Line drawings of nuclear boundaries were taken from a standard atlas that was then warped to align with the structural MRIs and microelectrode mapping results from individual animals. GPi, globus pallidus-internus; SCP, superior cerebellar peduncle; VLa, ventrolateral anterior nucleus; VLp, ventrolateral posterior nucleus. (TIF) [file pbio.3000829.s001.tif]

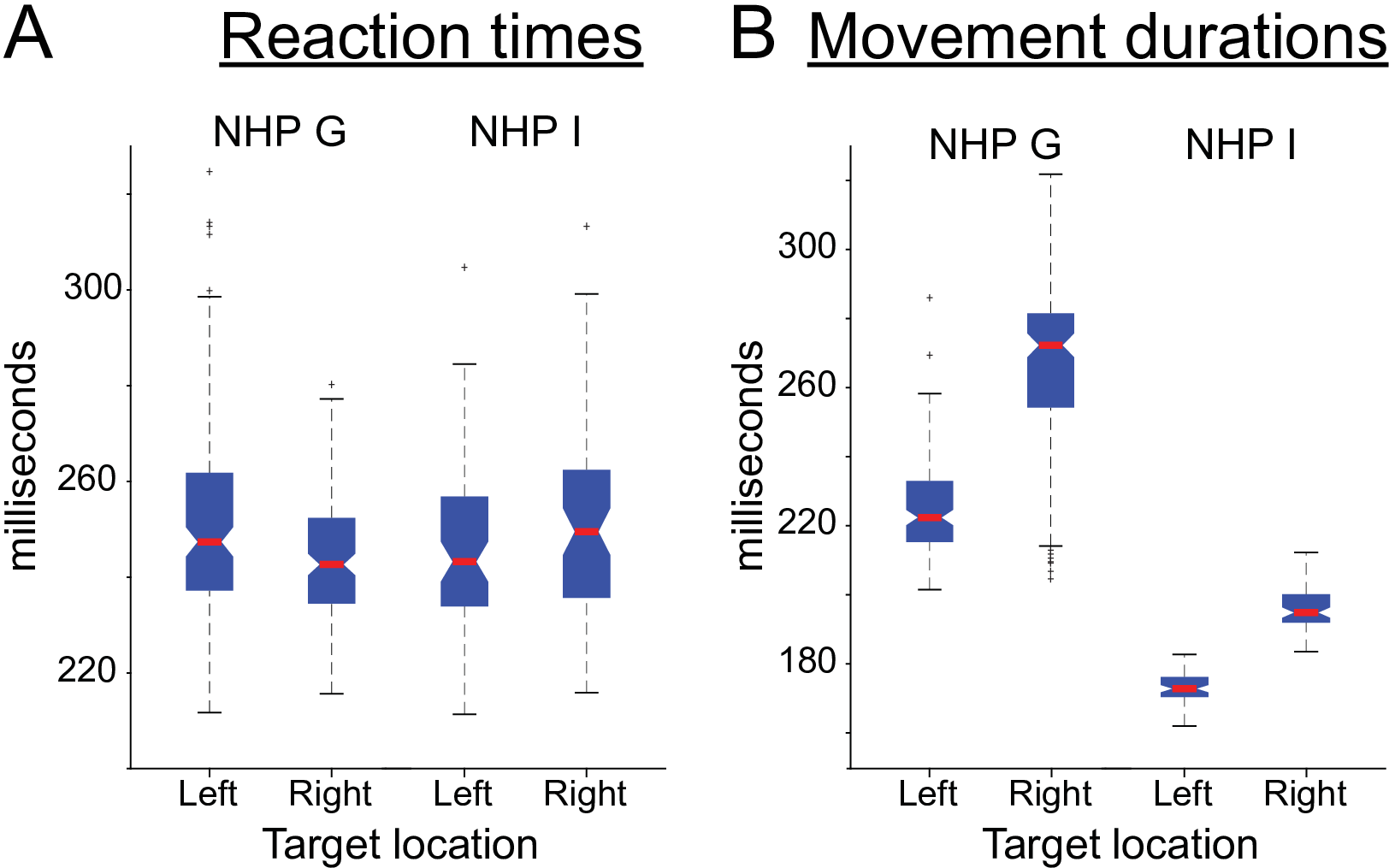

Supplement: S2 Fig — (A) Reaction times did not differ significantly between the two animals (NHP G versus NHP I) or between the two reach directions (left versus right target location). (B) Movement durations were longer for reaches to the right target than to the left target. NHP G moved more slowly overall compared with NHP I. Data and code to reproduce this figure can be found in https://doi.org/10.5061/dryad.0cfxpnvxm (FigS2.m). NHP, nonhuman primate. (TIF) [file pbio.3000829.s002.tif]

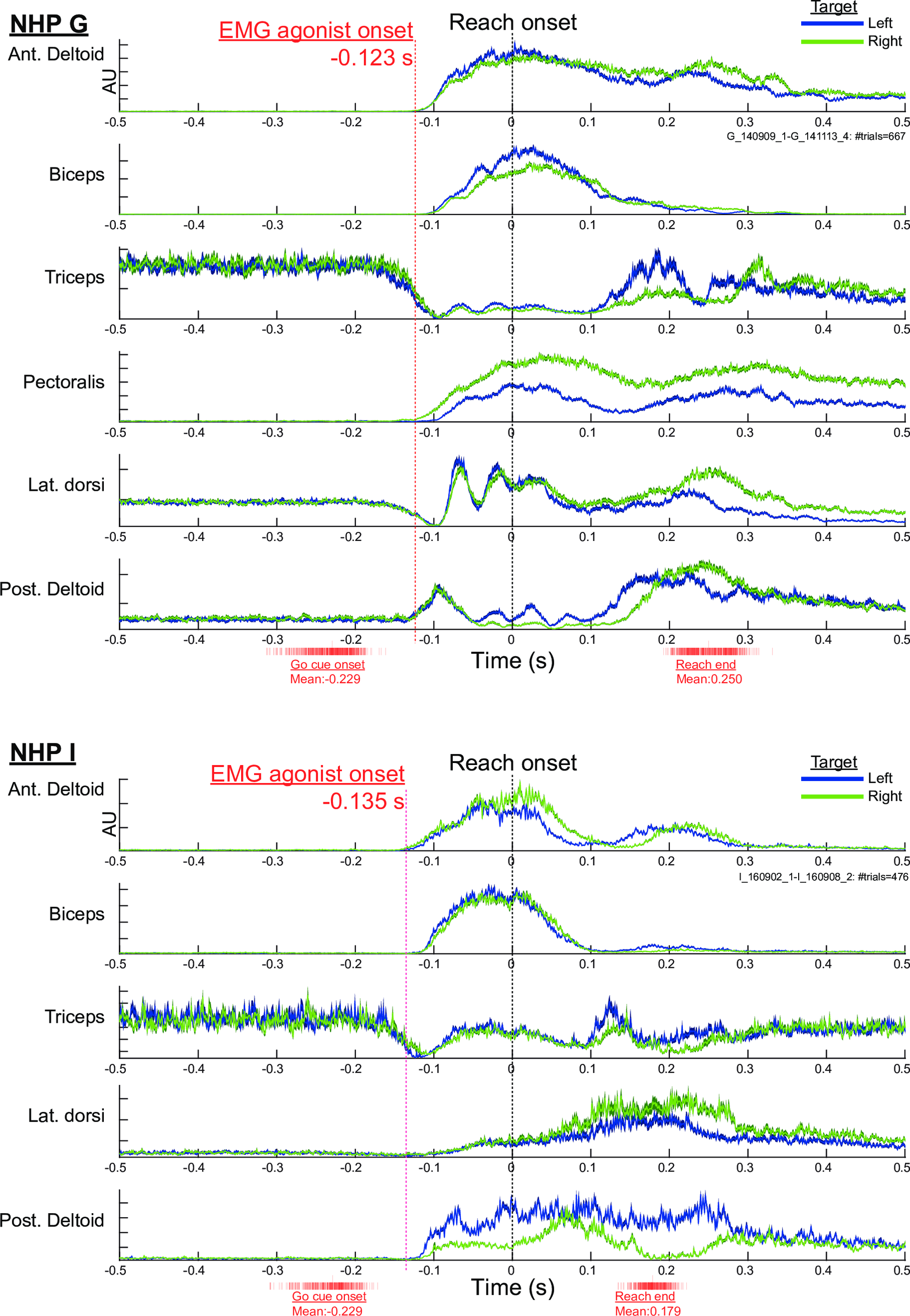

Supplement: S3 Fig — Rectified low-pass filtered EMG from proximal arm muscles was collected during a subset of data collection sessions (n = 667 and 476 trials in NHPs G and I, respectively). Signals from each muscle were averaged across trials separately for reaches to left and right targets (blue and green traces, respectively). The width of each trace reflects the SEM. The earliest reach-related modulation in EMG in both animals consisted of a reduction in triceps resting activity followed soon thereafter by increases in two agonist muscles (anterior deltoid and biceps; red vertical dashed lines), which occurred at similar premovement timing in both animals (−123 and −135 ms in NHPs G and I). For reference, red tick marks at the bottom of each panel indicate the times on individual trials of go-cue presentation and target touch at the end of the reach. EMG from pectoralis was not available for NHP I because of poor signal quality. Data and code to reproduce this figure can be found in https://doi.org/10.5061/dryad.0cfxpnvxm (FigS3.m). EMG, electromyography; NHP, nonhuman primate; SEM, standard error of the mean. (TIF) [file pbio.3000829.s003.tif]

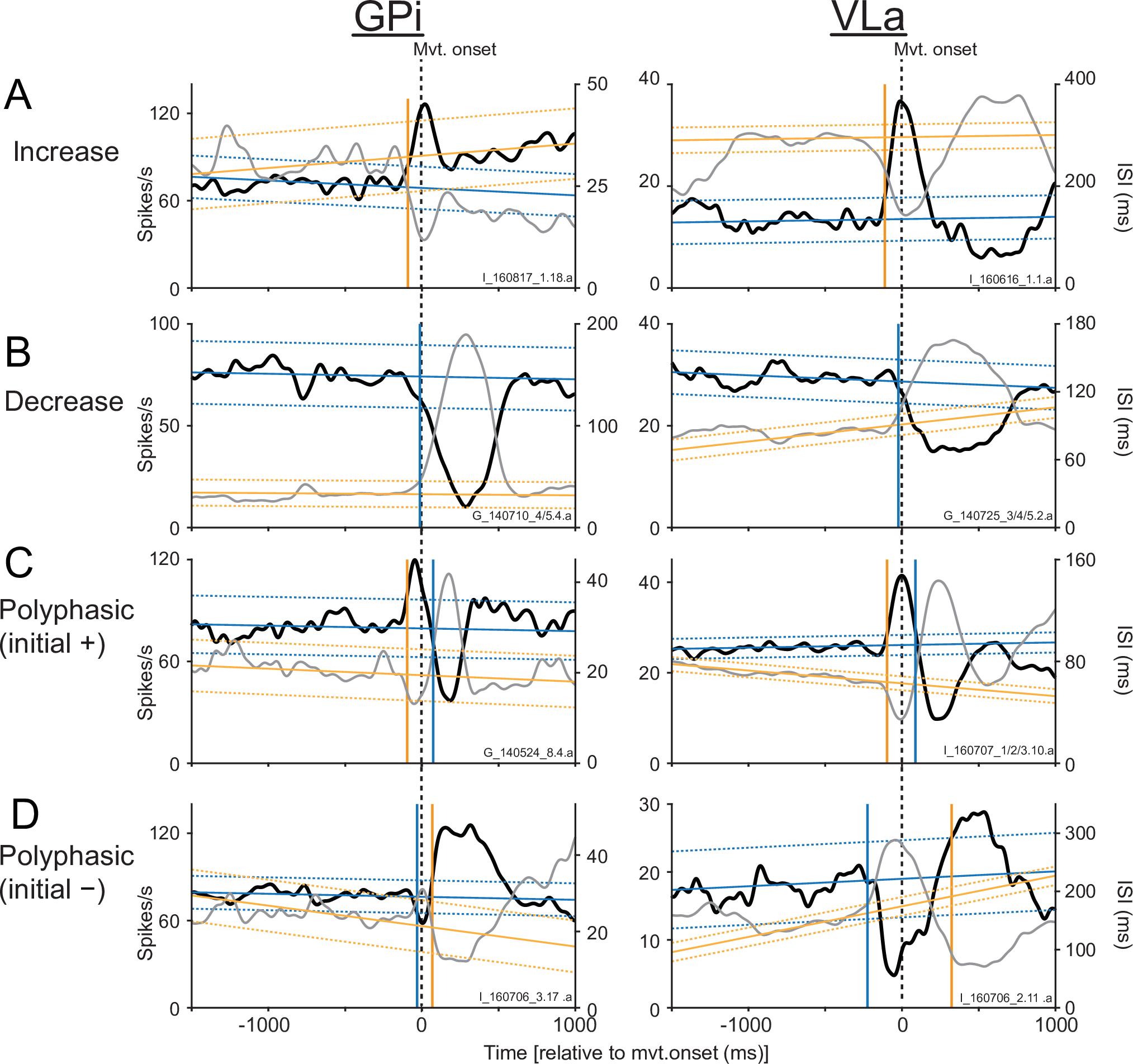

Supplement: S4 Fig — The panel for each single unit shows, in overlay, a mean spike-density function (black, left y-axis) and a mean interspike-interval function (gray, right y-axis), both constructed from the same underlying spike train. The spike-density function was used to test for increases in firing rate relative to pre–go cue baseline activity (linear trend ± CI, sloped yellow lines solid and dotted, respectively). The onset time of significant increases in firing are indicated by vertical yellow lines. The interspike-interval function was used to test for peri-movement decreases in firing, again relative to baseline activity (linear trend ± CI, sloped blue lines). The time of onset of significant decreases in firing are indicated by vertical blue lines. Note the presence of significant increases and decreases in firing for single units with activity classified as polyphasic. Data and code to reproduce this figure can be found in https://doi.org/10.5061/dryad.0cfxpnvxm (FigS4.m). GPi, globus pallidus-internus; VLa, ventrolateral anterior nucleus. (TIF) [file pbio.3000829.s004.tif]

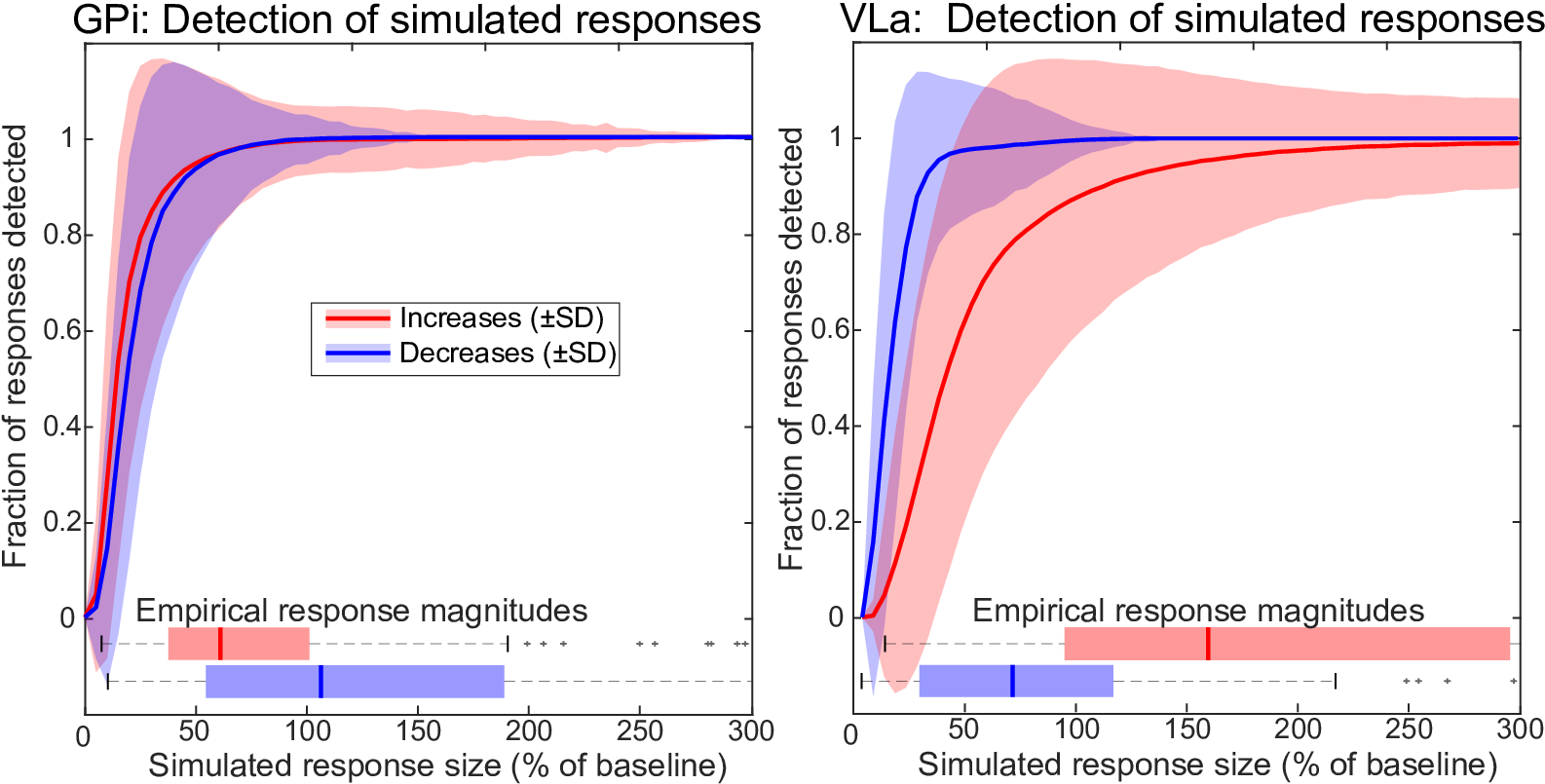

Supplement: S5 Fig — Curves reflect the mean (±SD) fraction of increases and decreases detected as a function of the simulated response size (expressed as percent of baseline firing rate). For GPi, the fraction of simulated responses detected was very similar for increases and decreases (red and blue curves, respectively) independent of the response size. For VLa, the algorithm was more effective at detecting decreases than increases when the simulated change in rate was relatively small (<100% of baseline). That bias disappeared for larger response sizes (>100% of baseline). Box plots show the distributions of response in our recorded data plotted separately for increases and decreases for each cell type. Data and code to reproduce this figure can be found in https://doi.org/10.5061/dryad.0cfxpnvxm (FigS5.m). GPi, globus pallidus-internus; VLa, ventrolateral anterior nucleus. (TIF) [file pbio.3000829.s005.tif]

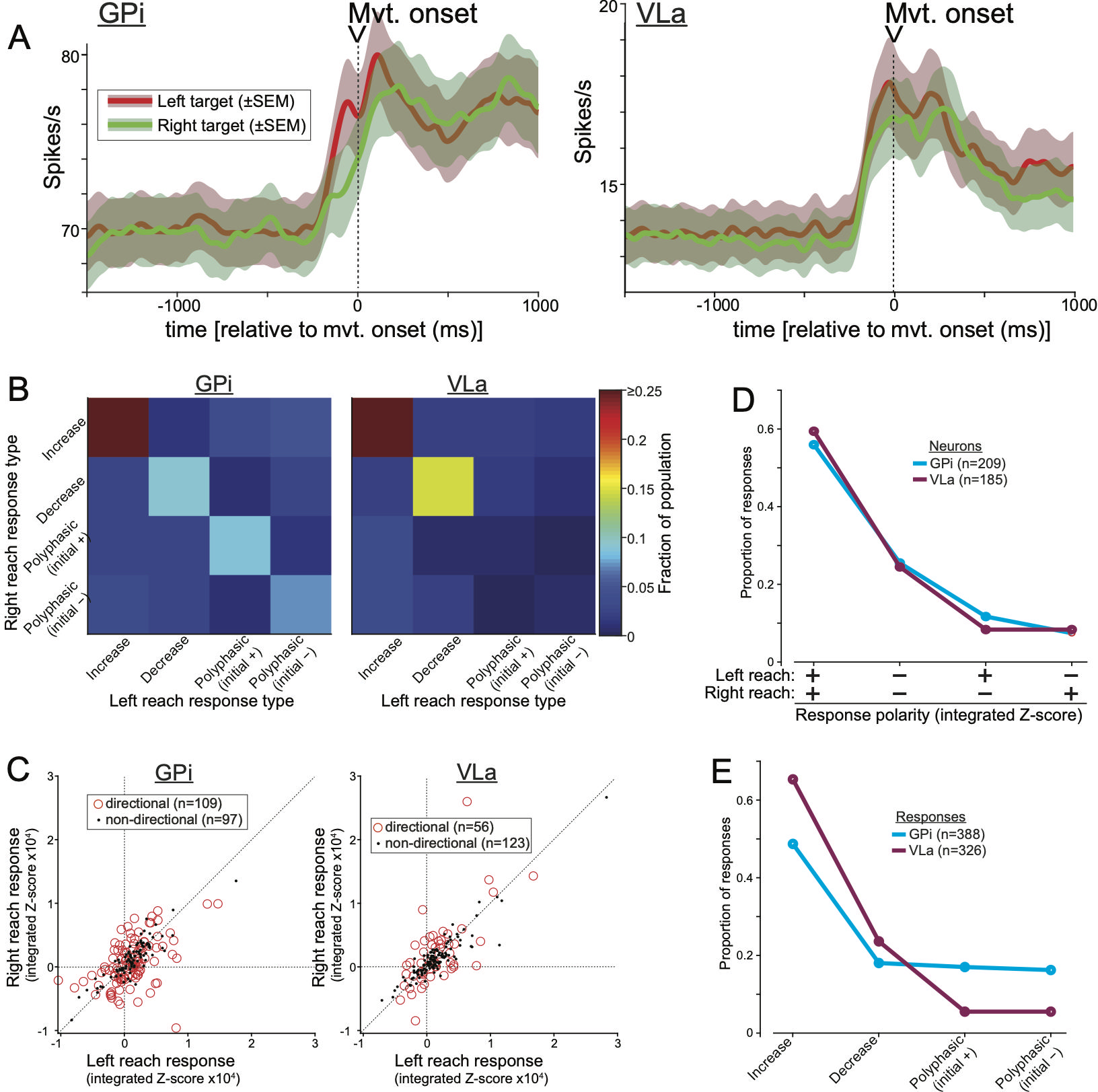

Supplement: S6 Fig — (A) Population-level peri-movement activity did not differ between directions of movement. Population mean spike-density functions (±SEM) compiled separately for movements to left and right targets. (B) The distribution of all possible pairings of the four response types in individual neurons for left and right directions of movement. Large fractions of neurons in GPi and VLa (color scale) had the same type of response for both directions of movement (denoted by colors in squares located on the diagonals of the matrices). (C) Integrated changes in firing rate during the peri-movement period plotted for individual neurons for movements to left (abscissa) versus right (ordinate) targets. The polarity and magnitude of integrated changes was in general correlated between the two directions for both GPi and VLa units. Nonetheless, the peri-movement activity of many units differed significantly between left and right reaches (red symbols; 53% and 31% of units in GPi and VLa, respectively). (D) The polarities of integrated firing-rate changes for left- and rightward movements were distributed similarly for neurons in GPi and VLa. (E) The overall proportions of peri-movement responses classified into the four response forms. Although the exact proportions differed somewhat between GPi and VLa populations, the overall distribution showed a similar pattern for GPi and VLa populations. Data and code to reproduce this figure can be found in https://doi.org/10.5061/dryad.0cfxpnvxm (FigS6_S8.m). GPi, globus pallidus-internus; SEM, standard error of the mean; VLa, ventrolateral anterior nucleus. (TIF) [file pbio.3000829.s006.tif]

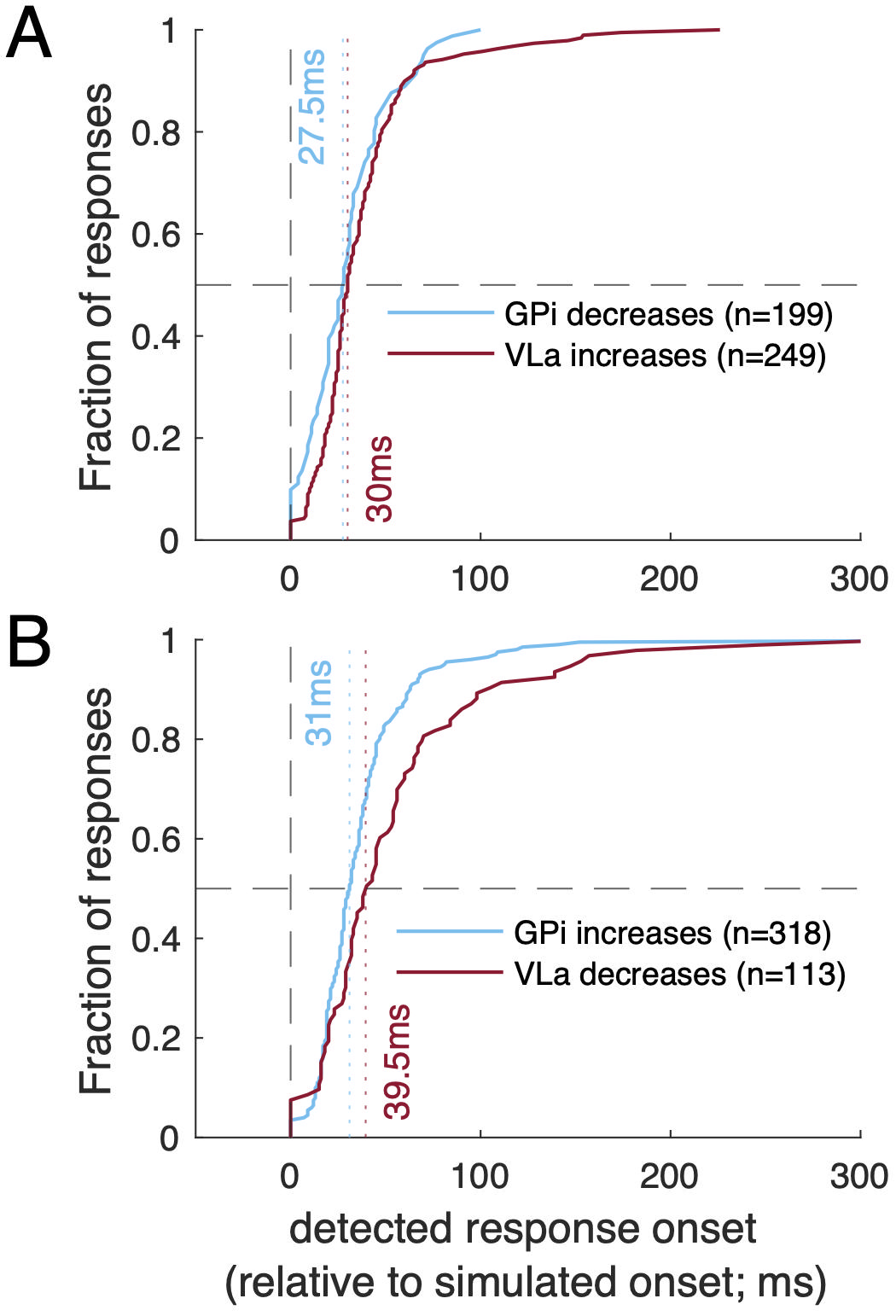

Supplement: S7 Fig — By design, the simulated responses began at time zero but otherwise matched the individual metrics of each empirically observed response (baseline rate, baseline variability, response magnitude, and slope of response onset). The figure follows the conventions of Fig 3. Detected onset times in GPi and VLa did not differ significantly. Data and code to reproduce this figure can be found in https://doi.org/10.5061/dryad.0cfxpnvxm (FigS7.m). GPi, globus pallidus-internus; VLa, ventrolateral anterior nucleus. (TIF) [file pbio.3000829.s007.tif]

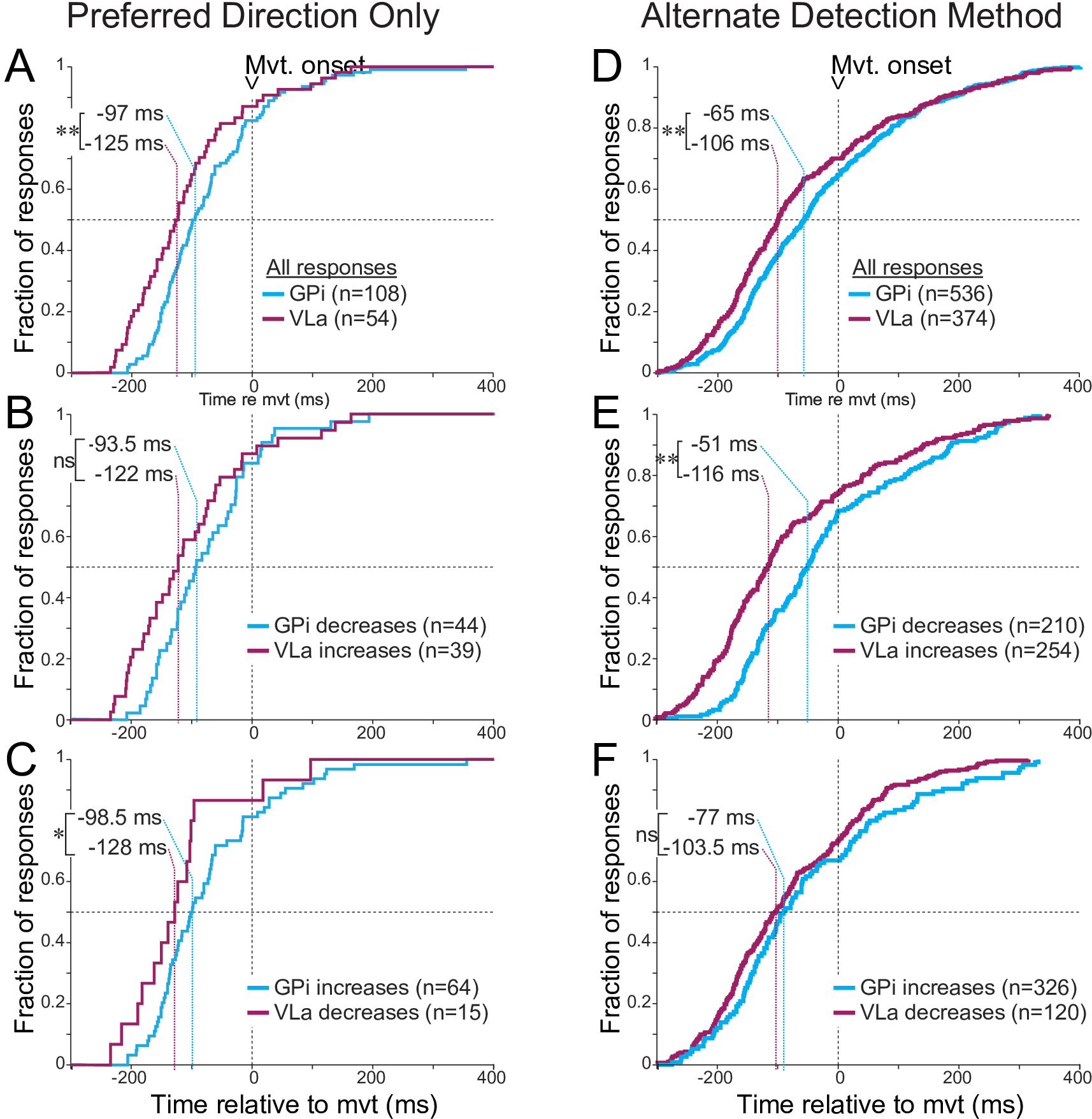

Supplement: S8 Fig — (A-C) Cumulative distributions of onset latencies restricted to responses in the preferred direction of units with peri-movement activity that was significantly directional. (D-F) Cumulative distributions of onset latencies as defined by the alternate, 10% of maximum, method. The figure follows the conventions of Fig 3. (A) Comparisons of latencies of all peri-movement changes detected in GPi neurons (blue) and VLa neurons (purple). VLa responses precede GPi by a median of 28 ms (**p < 0.001 rank sum test). (B) Response onset latencies of VLa increases (purple) lead GPi decreases (blue) by a median of 28.5 ms (ns p > 0.05 rank sum test). (C) Response onset latencies of VLa decreases (purple) lead GPi increases (blue) by a median of 29.5 ms (*p < 0.05 rank sum test). (D) Comparisons of latencies of all peri-movement changes detected in GPi neurons (blue) and VLa neurons (purple). VLa responses precede GPi by a median of 41 ms (**p < 0.001 rank sum test). (E) Response onset latencies of VLa increases (purple) lead GPi decreases (blue) by a median of 65 ms (**p < 0.001 rank sum test). (F) Response onset latencies of VLa decreases (purple) lead GPi increases (blue) by a median of 13.5 ms (ns p > 0.05 rank sum test). Data and code to reproduce this figure can be found in https://doi.org/10.5061/dryad.0cfxpnvxm (FigS6_S8.m, Fig2_3_S8_S9.m). GPi, globus pallidus-internus; ns, not significant; VLa, ventrolateral anterior nucleus. (TIF) [file pbio.3000829.s008.tif]

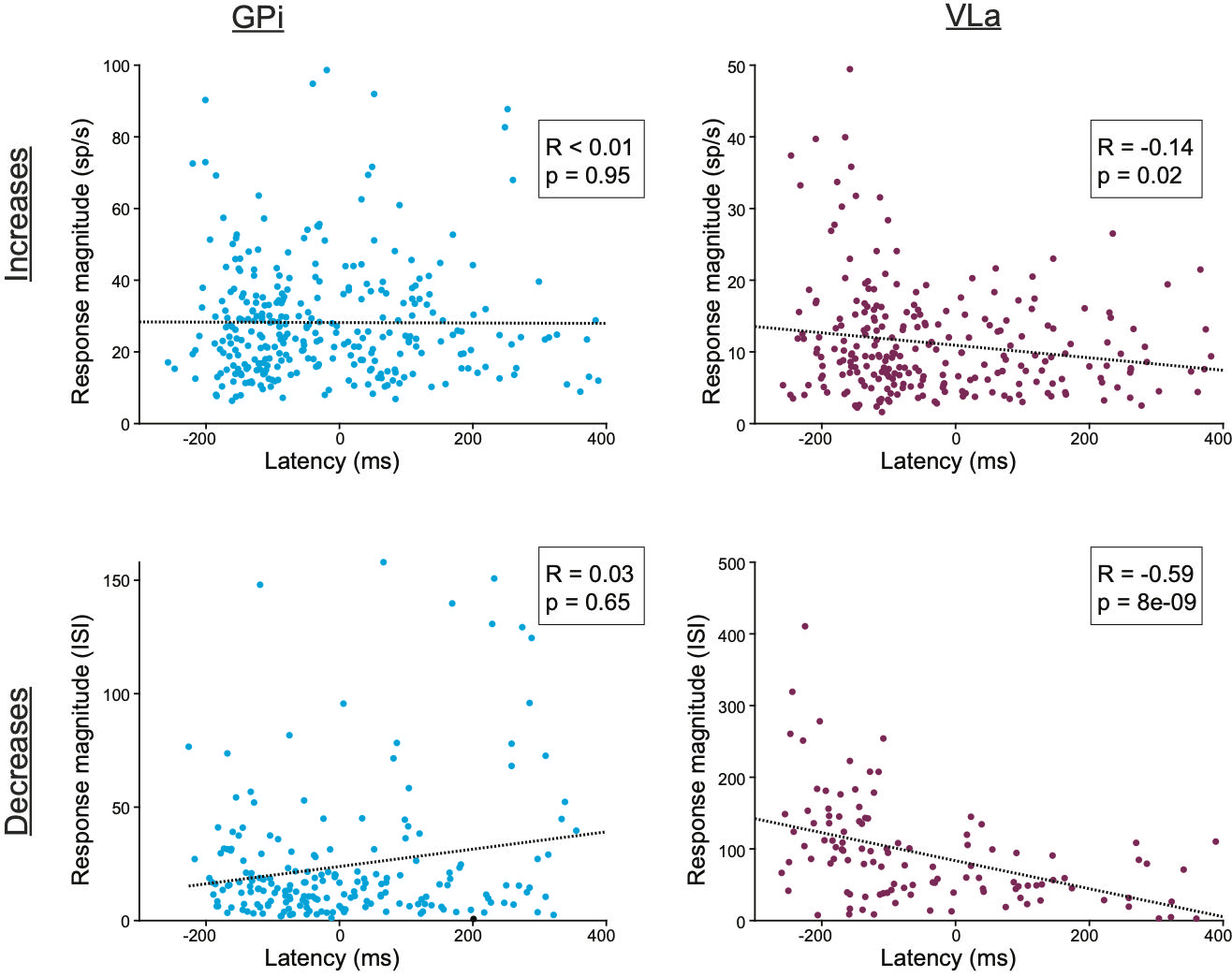

Supplement: S9 Fig — In GPi (blue), response magnitudes did not differ significantly as a function of onset latencies for either increase- or decrease-type responses (top and bottom scatterplots, respectively). In VLa (purple), response magnitudes were larger for early-onset responses than for late responses. Insets: values for rho and significance from Spearman rank correlation tests. Data and code to reproduce this figure can be found in https://doi.org/10.5061/dryad.0cfxpnvxm (Fig2_3_S8_S9.m). GPi, globus pallidus-internus; VLa, ventrolateral anterior nucleus. (TIF) [file pbio.3000829.s009.tif]

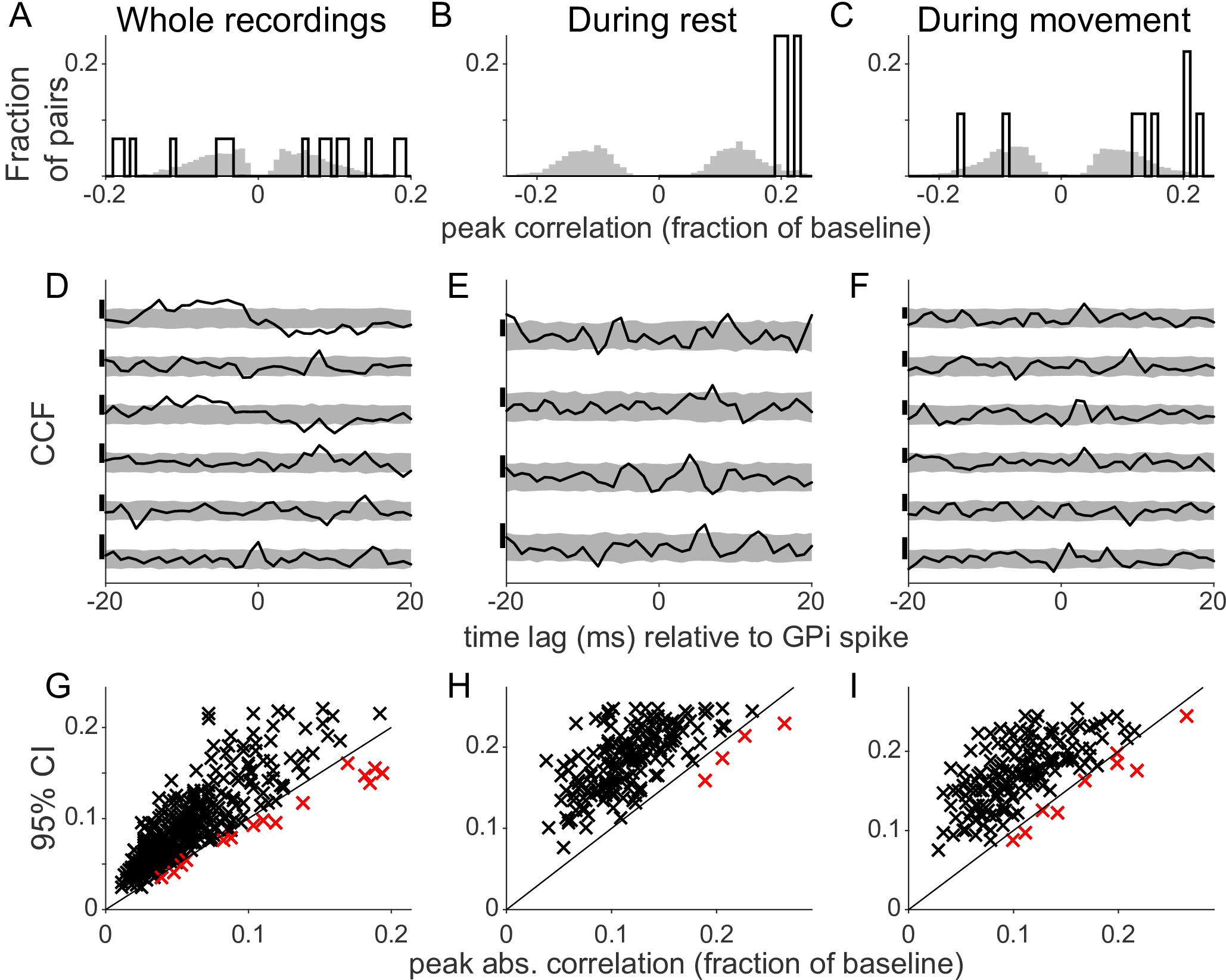

Supplement: S10 Fig — (A-C) Histograms of significant signed peak correlations. (D-F) Example CCFs achieving significance. Vertical scale bars for each example indicates CCF = 0.2. (G-I) Scatterplots of peak correlation values and confidence intervals. Data and code to reproduce this figure can be found in https://doi.org/10.5061/dryad.0cfxpnvxm (Fig4_S10to13.m). CCF, cross-correlation function. (TIF) [file pbio.3000829.s010.tif]

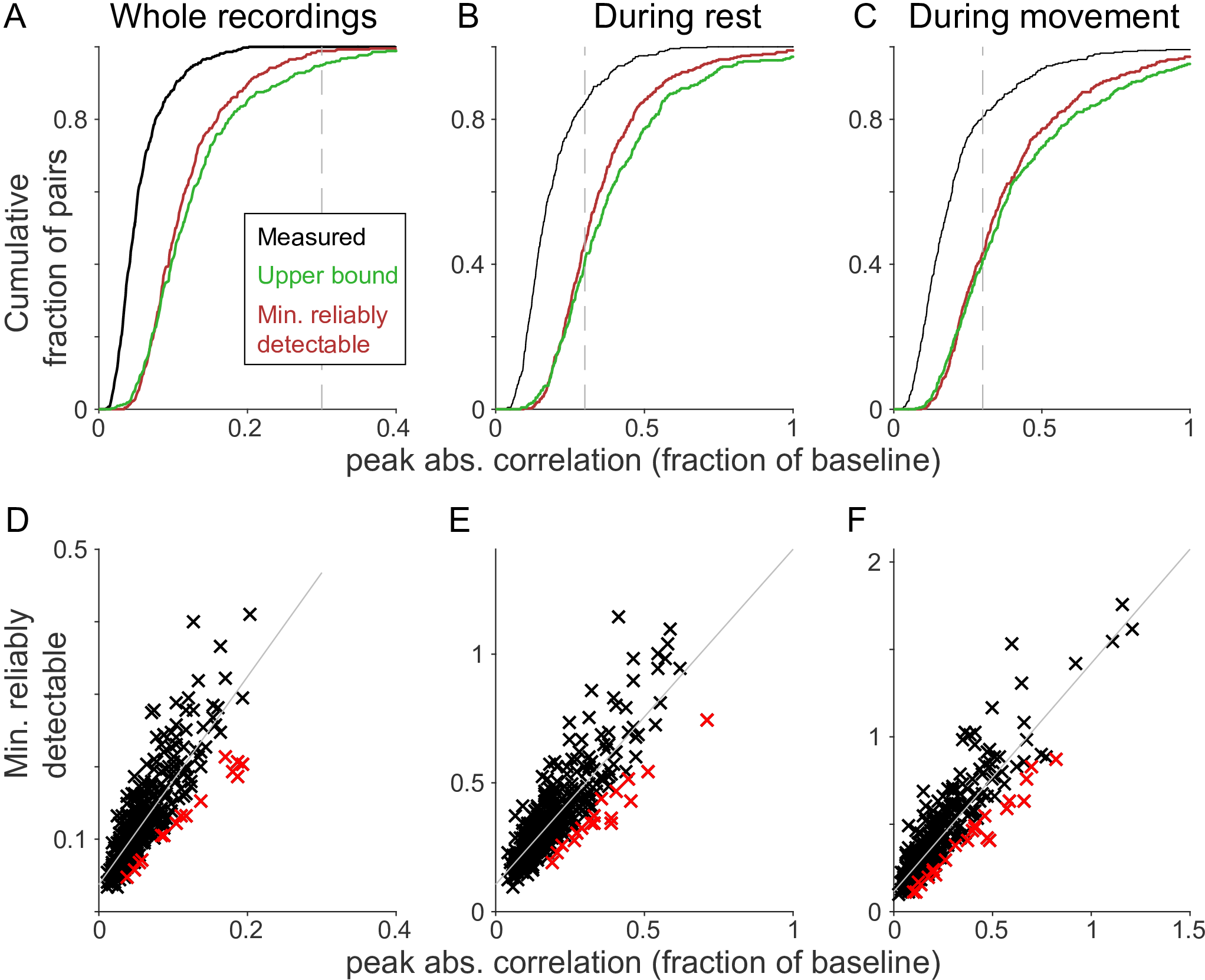

Supplement: S11 Fig — (A-C) Cumulative histograms of measured peak correlations (black) before exclusion of pairs with c0>0.3, minimum reliably detectable correlations c0 (red), and upper bounds of the measured correlations cu (green). The cutoff co>0.3 is indicated by dashed lines. (D-F) Scatterplots of measured correlations versus co. Significant correlations are marked red. Data and code to reproduce this figure can be found in https://doi.org/10.5061/dryad.0cfxpnvxm (Fig4_S10to13.m). CCF, cross-correlation function. (TIF) [file pbio.3000829.s011.tif]

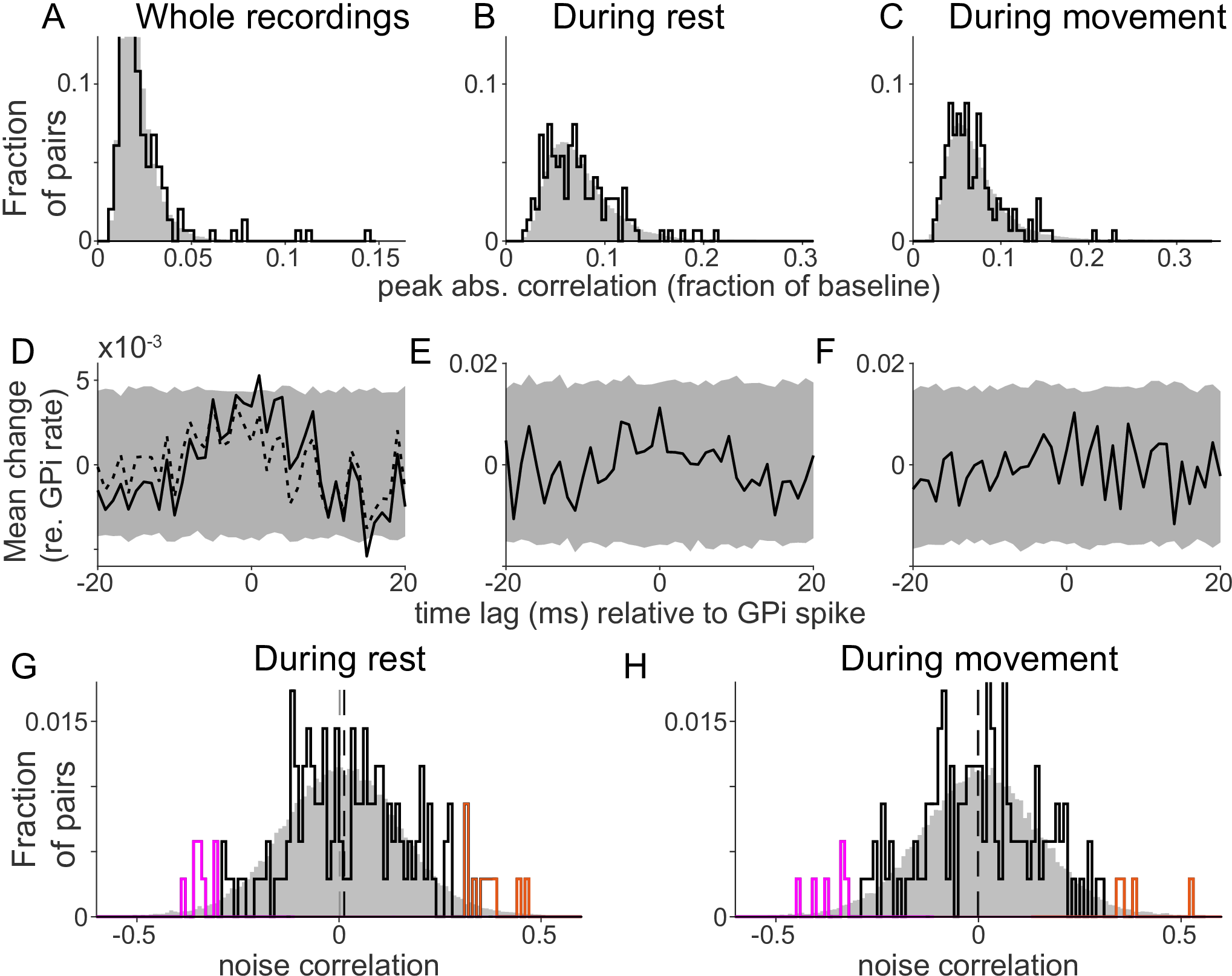

Supplement: S12 Fig — The figure follows the conventions of Fig 4. Dashed lines in (D) indicate the average CCF after exclusion of outliers. Data and code to reproduce this figure can be found in https://doi.org/10.5061/dryad.0cfxpnvxm (Fig4_S10to13.m). CCF, cross-correlation function; GPi, globus pallidus-internus. (TIF) [file pbio.3000829.s012.tif]

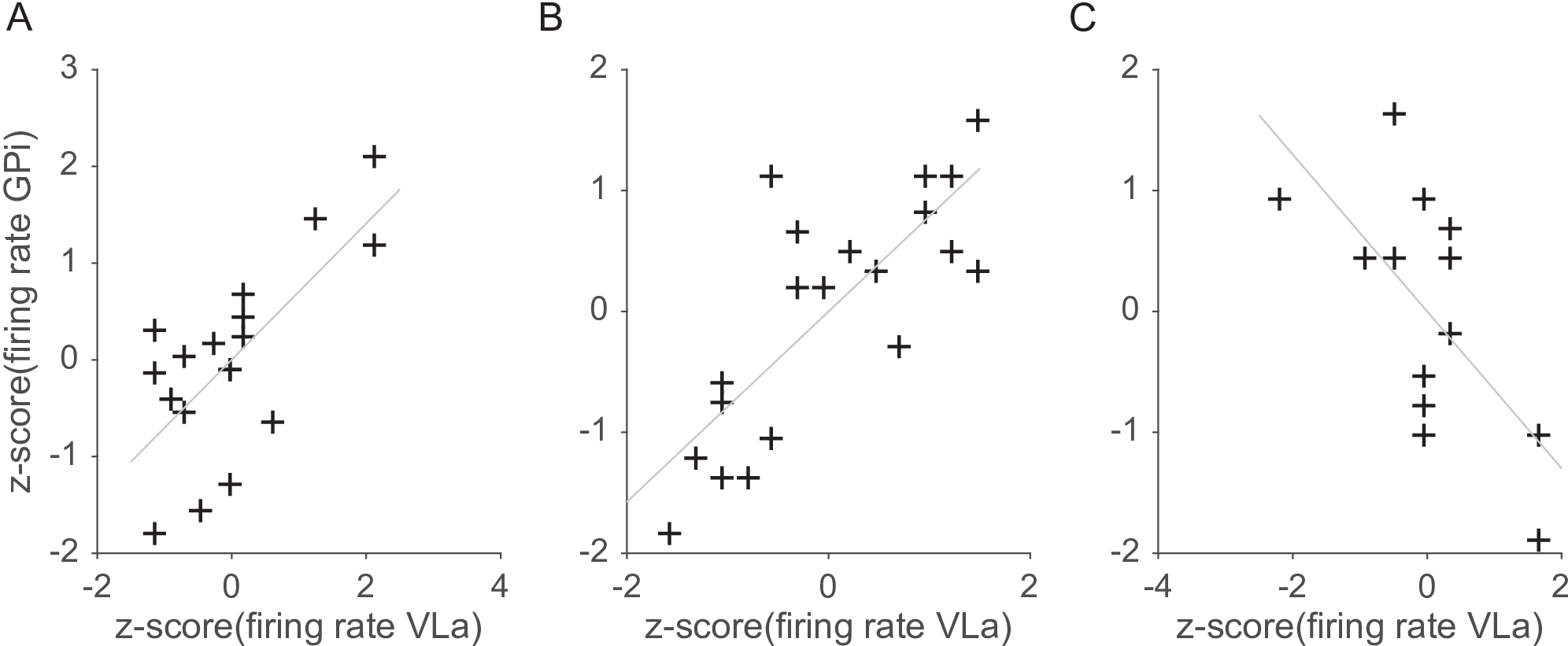

Supplement: S13 Fig — Data and code to reproduce this figure can be found in https://doi.org/10.5061/dryad.0cfxpnvxm (Fig4_S10to13.m). GPi, globus pallidus-internus; VLa, ventrolateral anterior nucleus. (TIF) [file pbio.3000829.s013.tif]

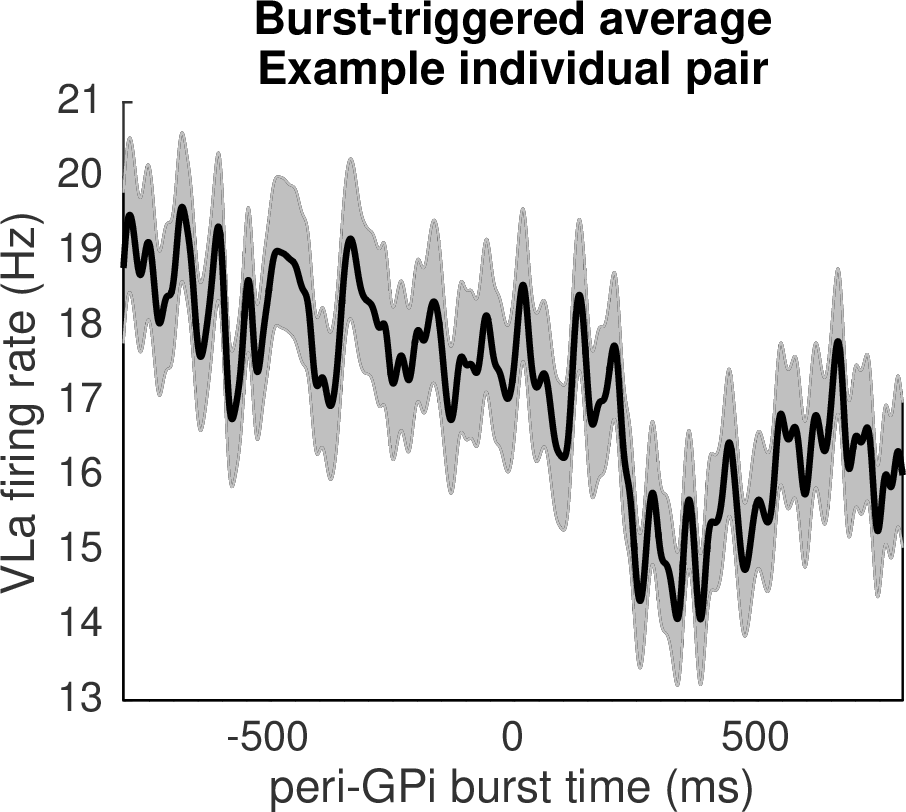

Supplement: S14 Fig — Burst offsets correspond to time zero. Data and code to reproduce this figure can be found in https://doi.org/10.5061/dryad.0cfxpnvxm (Fig5_S14.m). GPi, globus pallidus-internus; SEM, standard error of the mean; VLa, ventrolateral anterior nucleus. (TIF) [file pbio.3000829.s014.tif]
