# Supplementary material for: Neural activity during a simple reaching task in macaques is counter to gating and rebound in basal ganglia–thalamic communication
Source: PLoS Biol. 2020 Oct 13;18(10):e3000829. doi: 10.1371/journal.pbio.3000829 (PMC7584254; doi:10.1371/journal.pbio.3000829)
Supplement: Attachment — Submitted filename: response_to_reviewers_final.docx [file pbio.3000829.s015.docx]

We are grateful for the thorough, constructive, and valuable comments that the reviewers have provided, and we highly appreciate their help in improving our manuscript.

Below, we document our detailed responses to the reviewers’ comments.

*Reviewer #1: This study examined the relationship between basal ganglia output and activity in the ventral thalamus in monkeys performing a reaching task. The authors tested currently popular hypotheses including gating and rebounding accounts, and found that these cannot explain the experimental data. Connected pairs of GPi and VLa neurons were analyzed and it was found that neurons in both areas tend to increase firing at the time of movement, and VLa activity often precedes GPi activity.contrary to predictions of the gating and rebounding theories. This is a thoughtful and solid study. The experiments are carefully conducted and the discussion is scholarly and thorough. The results, while not entirely novel, are the first from identified GPi and VLs neurons in monkeys to my knowledge. For the most part, I find the data and arguments convincing, but I have a few suggestions.*

*1. In the old primate literature, there were claims that basal ganglia activity follows rather than precedes movement onset. This is not the case here. Could the authors comment on this issue? Does the current finding suggest that the basal ganglia may initiate movement?*

The reviewer mentions an important point which we did not discuss in the previous version of our manuscript. In our study, movement onset is defined as the earliest time point when the animal’s hand stops interrupting an infrared light beam. Activation of the antagonist muscles started approximately 130 milliseconds earlier than the detected onset of overt movement. We have added new supplementary information illustrating mean peri-movement electromyographic (EMG) activity for agonist and antagonist muscles in the two animals. Methods for EMG recording and analysis have been added. And, the following text has been added to the Discussion:

“It is worth noting, however, that our latency results, in which GPi responses began largely after the onset of agonist EMG, agree with many previous studies that used a wide variety of motor tasks performed in NHPs [Mink & Thach, 1991; Anderson & Horak, 1985; Georgopoulos et al., 1983; Turner & Anderson, 1997] and in rodents [Meyer-Luehmann et al. 2002].”

*2. GPi also projects to brainstem/midbrain areas. What is the role of these target regions in the reaching movement? Do the authors expect a different pattern of results? Would gating or rebound be more relevant in the non-thalamic targets?*

We appreciate the possible importance of this projection, which we neglected to mention in the previous version of our manuscript. We have added text to remedy this omission in the Introduction (pg. 6), but since we did not study this pathway in this work, we have kept that text brief:

“Other downstream targets of the BG, including areas of the brainstem and midbrain [Rye et al., 1988; Mena-Segovia et al., 2004], are also of great importance but are not investigated here.”

As we did not record from brainstem/midbrain, we can only speculate on the influence of this connection. For example, if convergence is lower for the pathway to brainstem/midbrain, the inhibitory influence of GPi may be stronger here (see our model, Fig. 6). Depending on intrinsic states of the brainstem/midbrain structures, gating or rebound may be possible. Unit recordings of these areas, ideally paired with unit recordings of basal ganglia output, are needed to clarify this question.

*3. Movement direction does not appear to modulate the GPi/VLa activity studied here. But previous work has shown strong effects of movement direction in basal ganglia. For example, more recent work (e.g. Barter et al 2015) showed that in rodents there are antagonistic cell groups defined by movement direction, and that basal ganglia specify instantaneous position coordinates. Also, might movmenets other than reaching explain some of the results here, for example small head movements.*

The description of our results may have been slightly misleading here. We did find direction selectivity in many of the recorded neurons (see Supporting Information S5 C: Red circles indicate significant directionality), although it was not strong. We tried to clarify this in the revised manuscript (pg. 14):

“While the majority of GPi neurons (109 out of 206) and some VLa neurons (56 out of 179) showed significantly stronger responses to one of the movement directions, […].”

Nevertheless, our main results did not depend on directionality (Supporting Information S6) and in only a minor fraction of neurons, the sign of responses changed depending on direction (Supporting Information S6 C, see also comments of reviewer #2).

We now also added a paragraph to the Discussion on the rodent work mentioned by the reviewer (Barter et al., 2015), which found antagonistic cell groups defined by movement direction (pg. 29-30):

“The qualitative form of peri-movement modulations in discharge as well as their timing were comparable between movement directions. Restricting analyzes to the preferred direction of each neuron did not change our main findings, stressing the absence of evidence for direction-selective gating or rebound. Compared to rodents [Barter et al., 2015], the general direction-selectivity of neurons in the basal ganglia recorded here was rather weak. That discrepancy may relate to differences in the tasks used for rodents and our NHPs: rodent tasks often [Barter et al., 2015; Schmidt et al., 2013] (but not always [Yttri & Dudman, 2016; Guo et al., 2017]) require large movements of the body in opposing (e.g., left vs. right) directions. In contrast, our NHPs performed discrete reaches with one arm toward one of two targets that were positioned only 14 cm apart such that muscle activity for those two reach directions differed only quantitatively (S3 Fig.). We cannot rule out the possibility that a task that required selection between categorically different movements would have yielded results more consistent with gating or rebound hypotheses.”

Some minor additional movements may have been present and could in general contribute to neural activity. However, it seems unlikely that such additional movements dominated activity observed here, as activity patterns were consistent across many trials, while additional spontaneous movements only occurred in few trials.

*4. There is strong agreement between current findings and that by Goldberg and Fee in songbirds. the model proposed by Goldberg and Fee suggested that high frequency entrainment occurs at high levels of glutamatergic inputs when pallidal 'pauses' are no longer necessary for thalamic spiking. What are the relevant candidates for this strong glutamatergic drive? The authors briefly mention 'a third source,' but perhaps more discussion of this issue could be helpful.*

We agree that the question of common drive to both GPi and VLa is important and merits more attention in our manuscript. In the absence of paired cortical recordings, and following suggestions of reviewer #2, we now include a description of anatomy as the basis for our speculation on a shared excitatory drive from cortex to both GPi and VLa in the Discussion (pg. 34):

“Given the lack of strong gating or rebound, how can we explain co-activation of GPi and VLa with movement? Rodent layer 5 pyramidal tract neurons of motor cortical areas innervate both BG and BG-recipient thalamus [Jeong et al., 2016; Prasad et al., 2020]. Thus, excitatory drive from motor cortex at with different delays may explain our observation of strong movement-related modulations in firing rate in both GPi and VLa without evidence for strong direct interactions between the two nuclei. Indeed, electrophysiological evidence indicates an efficient excitatory cortical control of motor thalamus [Bokor et al., 2016, Guo et al., 2017], possibly explaining the early activation of VLa with movement onset.”

*5. The following statement should be qualified, as there is no indication that inactivation or ablation is complete: "inactivation or ablation of BG outputs have, at most, minor detrimental effects on the performance of familiar motor tasks both in human patients [Svennilson et al. 33 1960; Baron et al. 1996; Cersosimo et al. 2008; Obeso et al. 2009] and in neurologically-normal non-human animals [Desmurget & Turner 2008; 2010; Piron et al. 2016; Horak & Anderson 1984; Inase et al. 1996].”*

We agree with the reviewer and changed the wording of this statement to (pg. 34):

“The present results are also consistent with the more general observation that inactivations or ablations placed in BG output nuclei have, at most, minor detrimental effects on the performance of familiar motor tasks both in human patients […]”

*Reviewer #2, Joshua Dudman: In the current submission the authors make a very detailed and careful analysis of recordings from the GPi (BG-output) and Vla (BG-recipient motor thalamus) to test what has been for ~30 years a canonical model to explain the role of basal ganglia in the function of forebrain motor circuits. Specifically, the idea that BG output disinhibits motor thalamus activity to select (by gating) a specific action remains a dominant model. However, as the authors note there have begun to be alternative models articulated. Although the data that underlie this dominant model are in fact rather limited and motivated primarily by relatively simple arguments (strong anatomical projection, high baseline activity of an inhibitory projection neuron, etc.). Nonetheless, I am sympathetic to the authors (implicit) belief here that a lot of careful analysis must be marshaled in order to rule out these existing models due to the persuasiveness of the simple arguments noted above. This paper is thus quite important in my mind because it is clearly the best and most careful assessment of the physiological predictions of such a model to date. I also should note that there is good reason such a comparison has not been made previously - it is very challenging to do well and the experiments here in my estimation have indeed been executed very well. My comments are primarily directed towards trying to improve the manuscripts accessibility to a broader audience that I fear could get occasionally lost in some more detailed points inspired by the very careful look being taken here.*

*Some general points that may be worth considering:*

*Although the text discusses other possible sources of common drive of motor thalamus and BG output as mysterious there is clearly some anatomy that can be described and cited to buttress the point and perhaps demystify a bit. Higher order thalamic nuclei like Vla are characterized by L5 and L6 feedback output from motor cortex. These L5 outputs to motor thalamus are pyramidal tract neurons that also project to multiple levels within BG. Thus, there is some clear anatomical basis for the possibility of a dominant, shared excitatory drive from cortex that could modulate both VLa and GPi.*

As already mentioned in the response to reviewer #1, we highly appreciate this suggestion and added the following paragraph to the discussion (pg. 34):

“Given the lack of strong gating or rebound, how can we explain co-activation of GPi and VLa with movement? Rodent layer 5 pyramidal tract neurons of motor cortical areas innervate both BG and BG-recipient thalamus [Jeong et al., 2016; Prasad et al., 2020]. Thus, excitatory drive from motor cortex at with different delays may explain our observation of strong movement-related modulations in firing rate in both GPi and VLa without evidence for strong direct interactions between the two nuclei. Indeed, electrophysiological evidence indicates an efficient excitatory cortical control of motor thalamus [Bokor et al., 2016, Guo et al., 2017], possibly explaining the early activation of VLa with movement onset.”

*At times I thought the key point that the absence of clear 'gating' like disinhibition does not mean that there is no functional consequence of this pathway got lost a little. It is returned to in the modeling and discussion at the end. However, if I interpret the data correctly this can be stated more succinctly in a few places. For example, at the end of the intro I thought a clearer statement about the resolution might be useful compared with:*

*" Our results suggest that, at least during performance of well-learned tasks in neurologically-normal animals, temporal influences of GPi outputs on VLa activity are subtle and that control over the timing and intensity of both pallidal and thalamic discharge may be dominated by other, possibly cortical, inputs."*

*I think this sentence highlights an unknown question raised by the data (I have more comments on this point below) whereas there is an important upshot to the results that gets a bit lost here. The authors show that the only way in which GPi output could have a very strong influence on activity in Vla is under the assumption of strong convergence of output projections and a highly correlated burst of activity across the GPi output population. Here, the authors provide strong evidence that in a typical reinforced task - reaching to one of two alternate targets - commonly thought to be BG dependent there is not such a strong correlated modulation of GPi output activity (either positive or negative) and that common drive to thalamus and BG dominate the observed activity. To my opinion that is a key positive take home from this paper that is in the discussion but might aid a reviewer by being moved up earlier in the text (here at the end of the intro) as well.*

We have reworded the end of the Introduction (pg. 6), although we do not wish to overstate our case for a specific alternative to gating and rebound:

“Hence, our results challenge the view that task-related activity in the BG-recipient thalamus arises under most or all conditions from gating or rebound-inducing signals transmitted from the BG. We use simulations to show how the strength of GPi-VLa communication can depend critically on the degree of anatomical convergence in the GPi-VLa circuit and the strength of spike synchrony within GPi. Thus, our results suggest that this pathway may function differently under other task conditions or behavioral states, in congruence with existing observations of effective thalamic inhibition by synchronized BG output.”

Also in the very last sentence of Discussion (pg. 37), we have now tried to make clear that we do not argue for the absence of any functional consequence of this pathway:

“Finally, subtle changes in BG output synchrony may be a potent way to scale the effectiveness of inhibitory drive to thalamus.”

As the reviewer points out, our simulations demonstrate that the influence of GPi on VLa is expected to depend on both convergence and GPi synchrony (with low convergence and high synchrony leading to strongest effects). We also further stress the possibility of a common cortical drive.

*I also wanted to note that there are some similar points that have been made about BG output in the past that are not referenced here. While I understand the emphasis on GPi and NHPs, the SNr has been studied a reasonable amount in rodents (and also projects to the homologous thalamic nuclei). Here too it was pointed out that due to a lack of correlated modulation it can appear that there is little effect of SNr projections, however, strong synchronous activation reveals a relatively potent effect of this inhibitory pathway, Brown 2014 eLife. This may provide additional support for the discussion about Kim 2017 optogenetic stimulation results and extracellular stimulation results discussed here. There has also in the past been data in NHP (Nevet 2007 JNP) showing little pairwise correlation amongst SNr neurons despite strong inhibitory synapses between these neurons further supporting points in this paper. (Although there is also evidence for strong unitary connectivity and effects on spike timing perhaps more akin to bird data in Goldberg in Higgs 2016 JNP as a counterpoint)*

We agree that the submitted version of the manuscript neglected the rodent literature a bit, and are happy to include the points mentioned by the reviewer (pg. 35):

“Also, rodent work has revealed a potent effect of synchronized compared to uncorrelated SNr activity [Brown et al., 2014]. […] Note that our observation of very low levels of between-neuron synchrony in GPi is consistent with multiple past studies in neurologically-normal animals [Bar-Gad et al. 2003b; Wilson 2013], even during performance of well-learned behavioral tasks [Nini et al. 1995; Wongmassang et al., 2020]. Similarly, the primary BG output nucleus in the rodent (SNr) shows similarly low levels of pairwise correlations [Nevet et al., 2007], potentially due to desynchronization by intrinsic inhibitory connections [Higgs & Wilson, 2016].”

*I thought in general the readability of the paper would be improved with some, even modest, description of the task that the monkeys are performing. It does appear in bits throughout the results, but for example, in this sentence " The mean firing rate of most single-units showed small but significant ramps during the start position hold period (i.e., before presentation of the task's go cue; p<0.05, linear regression; 97% and 98% of GPi and VLa cells, respectively)" the reader has no appreciation of what "start position hold period" is yet because there has only been a passing citation to describe the task. I felt that the general implications of the results could be easier to understand if the task we described (just a bit) and the predictions of the getting model clarified in that context.*

We now added a short description of the task to the first paragraph of Results (pg. 7):

“The spiking activity of isolated single-units was recorded from both areas simultaneously in macaque monkeys while the animals performed a two-choice reaction time reaching task for food reward [Franco & Turner 2012; Zimnik et al. 2015]. In short, animals were trained to hold their left hand at a home position located immediately to the left of the animal at waist height. Two visual targets were located 14 cm apart at shoulder height on a vertically-mounted response panel positioned 30 cm in front of the animal. After a randomized home position hold period, one of the targets (chosen pseudo-randomly trial-to-trial) was lit and the animal had to move the left hand swiftly to that target (distance: ~37 cm) and then hold there to obtain a reward. The right arm was restrained in a padded splint throughout a data collection session.”

We also clarified that movement directions in our experiment did not differ as strongly as movement directions in typical rodent tasks (pg. 29):

“In contrast, our NHPs performed discrete reaches with one arm toward one of two targets that were positioned only 14 cm apart such that muscle activity for those two reach directions differed only quantitatively (S3 Fig.).”

*" In summary, the general distribution of different response types and the predominance of firing-rate increases were similar in GPi and VLa (see Supporting Information S5 E), contrary to the prediction from the gating hypothesis that this relationship would be reciprocal."*

*The point that the gating model cannot explain the data is very clearly and strongly made with the existing data analysis. Yet, I might also point out one place that didn't receive much discussion which is S5C. The gating model would predict if a given GPi neuron is inhibited for say a R reach then it should be positively or null modulated for L reach ('selecting' by disinhibition the R reach). However, those two quandrants (<0 for one direction and >0 for other direction) have relatively little cells and does not capture the plurality of the data.*

We thank the reviewer for pointing to this additional evidence against gating, which we added to Results (pg. 15):

“Notably, few points fell in the upper left and lower right quadrants of Supporting Information S6 C reflecting the fact that few neurons were modulated reciprocally by the two movement directions or exclusively for one direction. Simple conceptions of the gating model predict that such relations with movement direction would be common in GPi.”

*Some minor points:*

*Very difficult to see red tick marks in Figure 2A*

Done

*Spikes/s (z-score) is a strange description for the colorbar*

The labels have been changed to “Normalized activity”

*The title for Figure 5D is confusing and could be clarified.*

We changed the title for Fig. 5D to: Start of pause vs. end of burst in GPi.

*In general the discussion is a little complicated to follow. Perhaps some version of a schematic interpretation of these observations could help a reader?*

We agree that it was challenging to follow our original Discussion and to obtain a big picture view of the findings and conclusions of our manuscript. To help the reader follow our arguments, we added Table 3 summarizing the expected outcomes of our analyses based on the assumptions of the gating and rebound theories as well as our observed results.

*I believe this is the wrong figure reference for this statement (should be S9) and the rest of the following paragraph " Contrary to the expectation that GPi-to-VLa CCF effects would be predominantly inhibitory (i.e., negative), roughly equal numbers of the CCF peaks detected as significant were positive and negative (8 versus 7, respectively; p=0.79, chi-square test; Supporting Information S8 D-F)." Subsequent paragraph should be S10 not S9 for simulation based estimation of correlation sensitivity. I don't have any substantive issues with the figures, I agree with their conclusions.*

We corrected all labels for the Supporting Information.

*Reviewer #3: In the present study, the authors aimed at understanding the mechanism by which the basal ganglia-thalamic (GPi-VLa) pathway communicates information. More precisely, they challenged two theories regarding the way GPi and VLa neurons interact during overlearned behavior, the gating and the rebound theories. These theories make several testable predictions, among which a strong temporal control of thalamic neurons by variations of GPi neuron activities. The authors also considered an alternative hypothesis, the "modulation" hypothesis for which a weaker relationship between task-related activities in GPi and VLa is required.*

*To this aim, the authors recorded and analyzed single-unit activities sampled (simultaneously or not) from connected areas of GPi and VLa in two rhesus monkeys over-trained to execute reaching movements toward two possible visual targets.*

*Using multiple and independent analyses, the authors found a majority of movement-related increases of activity in both GPi and VLa, earlier activity onsets in VLa compared to GPi, weak correlated activities in pairs of GPi and VLa cells, and weak effects of bursts or pauses of GPi neurons spontaneous activity on VLa responses. All these observations are not compatible with the predictions of the gating and/or rebound hypotheses. Finally, using simulations, the authors show that the strength of the GPi-VLa communication depends on the degree of anatomical convergence within the GPi-VLa circuit and on the strength of the spike synchrony within GPi.*

*Based on these experimental and simulation results, the authors propose that the temporal influence of GPi on VLa activity is weak and that responses in the two structure as well as communication between them might be under control of other inputs.*

*General comments*

*Overall, my opinion about the paper is positive. The study addresses a timely and important problem regarding how operations computed in the basal ganglia influence decision and action-related activity in the cortex, via the thalamus. Even if the study does not allow to definitely answer that question, it indicates that two influential views (the gating and rebound theories) are likely false, at least during overlearned behavior.*

*The authors very carefully designed their experiment, especially the methods necessary to control for the connection between the GPi and VLa areas in which neurons were recorded. They also show a very rigorous data analysis flow (including systematic control and redundant procedures as well as production of synthetic data to test their analysis algorithms). As a result, the conclusions of the authors appear very convincing and supported by the data. Moreover, limits and potential caveats are mentioned in the discussion section, which is always a positive point in a paper. The present work is thus very solid, important and well communicated via didactic writing.*

*I only have two relatively minor critics that I would nevertheless like to see addressed before publication.*

*First, the introduction directly starts with the problematic addressed by the authors (i.e. the connection between the basal ganglia and the thalamus). I believe that one or two broader sentences would help the reader to figure out why this question is important and need investigation. For instance, the authors state that "the connection between the basal ganglia (BG) and its major downstream target, the thalamus, has…" This sentence seems to indicate that the downstream pathway from the BG to the brainstem motor center is less important, which is not true.*

We agree on the importance of the pathway from the BG to the brainstem and adjusted the very first sentence of the Introduction (pg. 4):

“The connection between the basal ganglia (BG) and one of its major downstream targets, the thalamus, has received increased attention recently […]“

as well as later parts of the Introduction (pg. 6, see also comment of reviewer #1):

“Other downstream targets of the BG, including areas of the brainstem and midbrain [Rye et al., 1988; Mena-Segovia et al., 2004], are also of great importance but are not investigated here.”

*My other concern relates to the modeling work. I had the feeling that this modeling exercise was somehow disconnected from the rest of the paper. I had the opportunity to read a previous version of the paper posted on bioRxiv and this older version did not include the simulations. I thus suspect that the authors were encouraged to add these simulations (maybe to address Kim et al., 2017 apparent conflicting results), which is fine, but I would be more convinced if the connection between their experimental data and this approach was better motivated and the interpretation better described. For instance, it is not clear to me whether the simulation result (and more globally the whole paper) supports the Goldberg et al.'s hypothesis about a modulatory role of the BG on the thalamic activity. If results support the modulatory hypothesis, the title of the paper is misleading because it indicates that the three theories are not supported by the data.*

The hypothesis by Goldberg et al. about a modulatory role of the BG on thalamic activity is in line with our results. We therefore adjusted the title to:

“Neural activity during a simple reaching task in macaques is counter to gating and rebound in basal ganglia-thalamic communication”.

Moreover, we edited the abstract, the end of the Introduction (pg. 7), and the final line of the Discussion (pg. 37) to try to clarify the insight afforded by our simulations and better link it to the other results in the paper.

*Minor points*

**	Abstract: The sentence mentioning the simulation ("Instead, simulations…") sounds unclear, especially with respect to the rest of the abstract. A better explanation of the simulation and a clearer connection between the two approaches would improve the last part of the abstract in my opinion.*

We adapted the last part of the abstract to:

“These results appear incompatible with standard gating and rebound models. Still, gating or rebound may be possible in other physiological situations: Simulations show how GPi-VLa communication can scale with GPi synchrony and GPi-to-VLa convergence, illuminating how synchrony of basal ganglia output during motor learning or in pathological conditions may render this pathway effective. Thus, in the healthy state, basal ganglia-thalamic communication during learned movement is more subtle than expected, with changes in firing rates possibly being dominated by a common external source.”

**	Just like the abstract, I found that the part of the introduction mentioning the modeling work lacks clarity. Especially, the sentence "This insight reveals how our results may be brought into congruence with apparently-contradictory existing observations" does not make much sense to me at this stage of the paper. This is regrettable because the rest of the text is really well written and didactic.*

We tried to clarify the modeling work in the last part of the Introduction:

“Hence, our results challenge the view that task-related activity in the BG-recipient thalamus arises under most or all conditions from gating or rebound-inducing signals transmitted from the BG. We use simulations to show how the strength of GPi-VLa communication can depend critically on the degree of anatomical convergence in the GPi-VLa circuit and the strength of spike synchrony within GPi. Thus, our results suggest that this pathway may function differently under other task conditions or behavioral states, in congruence with existing observations of effective thalamic inhibition by synchronized BG output. Overall, our results suggest that, during performance of well-learned tasks in neurologically-normal animals, temporal influences of GPi outputs on VLa activity are subtle and at most modulatory. Control over the timing and intensity of both pallidal and thalamic discharge are therefore likely to be dominated by other, possibly cortical, inputs.”

**	Results - p 20: The authors refer to the supporting information S9 and not S8 as it is currently mentioned in the whole paragraph.*

We corrected that this paragraph refers to Supporting Information S10 (numbers changed as we added Supporting Information S3).

**	Results - p 21: The authors should have mentioned supporting information S10X instead of S9X. Actually, from supporting information S9, all references to supporting information are erroneous and should be corrected with n+1.*

We corrected all labels of the Supporting Information as correctly stated by the reviewer.

**	Results - p 24: It would be interesting to know whether or not the 119ms separating VLa long-lasting decrease of activity from GPi burst onset is compatible with transmission delays between these two areas.*

This is an important point. We now state that the long-lasting decreases in Fig. 6 are not compatible with short synaptic transmission delays between basal ganglia and thalamus (Discussion, pg. 32):

“Critically, those reductions in firing rate occurred at around 119ms after burst onset and were thus much slower than what would be expected for the synaptic transmission (compare to Fig. 1 B: VLa was inhibited few milliseconds after GPi stimulation).”

**	Results - p 26: The formulation "c(N-1)/N+1/N is not clear to me. Could the authors provide more details?*

In our simulations, observed correlations were approximately proportional to the quantity [c*(N-1)+1]/N, where c is the pairwise GPi correlation and N the number of converging GPi neurons. We wrote this as the sum of two terms to emphasize the linearity of the expression with respect to c (i.e., a line with slope (N-1)/N and intercept 1/N). This linear approximation is described in detail in Materials and methods --- Simulations (pg. 51-53). We have revised the expression on pg. 26 to read “[(N-1)/N]c + 1/N” so that it now appears in standard slope-intercept form.

**	Methods - p 37: How did the authors controlled monkeys' non acting hand?*

The right (non-acting) hand was mechanically restrained using a padded splint. We added this piece of information both at the start of Results (pg. 7) and to Materials and Methods (pg. 39).

**	Methods - p 39: In the paragraph entitled "Recording and stimulation protocol", the stimulation protocol is not described.*

We have corrected this mistake. The cited section was intended to describe only neuronal recording. The stimulation protocol was described in the previous sub-section titled “Localization of target regions for recording in GPi and VLa.” We have revised text in that section to make it more clear that electrical stimulation was performed during initial mapping experiments to identify target regions in GPi and VLa for subsequent paired recording. Details on the stimulation parameters used is provided in that section.

“Target region localization was then refined using single unit microelectrode recording in combination with electrical stimulation through electrodes in BCX and GPi (single biphasic pulses <200uA, 0.2ms-duration at 2Hz max.; Model 2100, A-M Systems; Fig. 1) and proprioceptive stimulation. The target region for recording in GPi was identified by the presence of typical high firing rate single-units, many of which responded briskly to proprioceptive stimulation of the forelimb [DeLong 1972; Turner & Anderson 1997] and responses to electrical stimulation in the arm region of primary motor cortex [Yoshida et al. 1993] (Fig. 1C). During localization of the target region in VLa, a macroelectrode was positioned acutely in the GPi. The target region for recording in VLa was identified by the presence of typical thalamic neuronal discharge that: a) responded to GPi stimulation with a short latency pause in firing [Anderson & Turner 1991] often followed by a rebound increase in firing probability; and b) did not respond to SCP stimulation, which would be indicative of a neuron in VLp, the cerebellar-recipient portion of motor thalamus located immediately posterior to VLa (Fig. 1B). Many VLa neurons also responded at short latency to stimulation in primary motor cortex. All subsequent data collection was directed to these target regions of GPi and VLa.”
